# BGP-15 Protects against Oxaliplatin-Induced Skeletal Myopathy and Mitochondrial Reactive Oxygen Species Production in Mice

**DOI:** 10.3389/fphar.2017.00137

**Published:** 2017-04-10

**Authors:** James C. Sorensen, Aaron C. Petersen, Cara A. Timpani, Dean G. Campelj, Jordan Cook, Adam J. Trewin, Vanesa Stojanovska, Mathew Stewart, Alan Hayes, Emma Rybalka

**Affiliations:** ^1^Centre for Chronic Disease, College of Health & Biomedicine, Victoria UniversityMelbourne, VIC, Australia; ^2^Australian Institute for Musculoskeletal ScienceMelbourne, VIC, Australia; ^3^Institute of Sport, Exercise & Active Living, Victoria UniversityMelbourne, VIC, Australia; ^4^Institute of Sustainability and Innovation, Victoria UniversityMelbourne, VIC, Australia

**Keywords:** skeletal muscle, oxaliplatin chemotherapy, BGP-15, mitochondria, protein synthesis, muscle wasting, mitochondrial reactive oxygen species

## Abstract

Chemotherapy is a leading intervention against cancer. Albeit highly effective, chemotherapy has a multitude of deleterious side-effects including skeletal muscle wasting and fatigue, which considerably reduces patient quality of life and survivability. As such, a defense against chemotherapy-induced skeletal muscle dysfunction is required. Here we investigate the effects of oxaliplatin (OXA) treatment in mice on the skeletal muscle and mitochondria, and the capacity for the Poly ADP-ribose polymerase (PARP) inhibitor, BGP-15, to ameliorate any pathological side-effects induced by OXA. To do so, we investigated the effects of 2 weeks of OXA (3 mg/kg) treatment with and without BGP-15 (15 mg/kg). OXA induced a 15% (*p* < 0.05) reduction in lean tissue mass without significant changes in food consumption or energy expenditure. OXA treatment also altered the muscle architecture, increasing collagen deposition, neutral lipid and Ca^2+^ accumulation; all of which were ameliorated with BGP-15 adjunct therapy. Here, we are the first to show that OXA penetrates the mitochondria, and, as a possible consequence of this, increases mtROS production. These data correspond with reduced diameter of isolated FDB fibers and shift in the fiber size distribution frequency of TA to the left. There was a tendency for reduction in intramuscular protein content, albeit apparently not via Murf1 (atrophy)- or p62 (autophagy)- dependent pathways. BGP-15 adjunct therapy protected against increased ROS production and improved mitochondrial viability 4-fold and preserved fiber diameter and number. Our study highlights BGP-15 as a potential adjunct therapy to address chemotherapy-induced skeletal muscle and mitochondrial pathology.

## Introduction

Cancer is a leading cause of world-wide mortality accounting for 8.2 million deaths in 2012 alone, with this figure predicted to reach 14 million by 2034 (World Health Organisation, 2015). In the majority of cases, first line treatment involves systemic chemotherapy administration. Chemotherapeutic agents target the molecular characteristics of cancerous cells, such as rapid replication, to chemically-induce cell death (de Gramont et al., 2000; Sorensen et al., 2016). However, due to its non-specific and systemic mode of action, chemotherapy also elicits effects on healthy tissues causing the classic side-effects attributable to anti-cancer therapy including nausea, vomiting, cardio-toxicity, immune disorders, peripheral and axial neuropathy, hair and weight loss and debilitative fatigue (Greene et al., 1993; Zitvogel et al., 2008; Gilliam and St Clair, 2011; National Cancer Institute, 2012; Ariaans et al., 2015). These side-effects often limit treatment tolerability, efficacy and therapeutic options, sometimes leading to the cessation of treatment all together and ultimately reducing patient quality of life and prognosis due to the development of co-morbidities (Gilliam and St Clair, 2011; Scheede-Bergdahl and Jagoe, 2013; Argilés et al., 2015; Cheregi et al., 2015). Emerging evidence suggests that the skeletal muscle is also a target of chemotherapy-induced atrophy (Pfeiffer et al., 1997), weakness and fatigue (Gilliam and St Clair, 2011), dysfunction (Scheede-Bergdahl and Jagoe, 2013; Bredahl et al., 2016) and insulin resistance (Ariaans et al., 2015). These effects appear to be more pronounced when chemotherapy is administered in childhood, due to the hyperplastic and hypertrophic nature of skeletal muscle at this early stage of life and persist well into adulthood (Ness et al., 2007; Scheede-Bergdahl and Jagoe, 2013; Ariaans et al., 2015). Since skeletal muscle has a high energy requirement, and thus a high mitochondrial density, emerging data suggests that skeletal muscle pathology may be underpinned by damage induced to the mitochondrial by chemotherapy administration (Davies and Doroshow, 1986; Doroshow and Davies, 1986; Sarosiek et al., 2013; Tabassum et al., 2015).

Oxaliplatin (OXA), a platinum (Pt)-based alkylating agent, is the leading anti-neoplastic agent against colorectal cancer (André et al., 2004; Gourdier et al., 2004; Aschele et al., 2005; Alcindor and Beauger, 2011), neuroblastoma (Tran et al., 2015) and solid tumors (Mascarenhas et al., 2013). Primarily, OXA elicits its antineoplastic effect by intercalating Pt adducts into the nuclear DNA (nDNA) causing single-stranded damage, cell cycle arrest and apoptosis (Alcindor and Beauger, 2011). In addition, it has been demonstrated that OXA has the capacity to induce a mitochondrially-driven apoptotic response that is independent of nDNA damage (Gourdier et al., 2004), implicating the mitochondria as inadvertent targets of OXA treatment. Preliminary findings by our laboratory demonstrate that OXA treatment induces significant mitochondrial dysfunction involving elevated mitochondrial (mt) reactive oxygen species (ROS) production and reduced viability of the mitochondrial pool *in vitro* (Cheregi et al., 2015). As hypothesized by us previously, these findings suggest that OXA explicitly damages the nDNA, but inadvertently hinders the mitochondria as well, by eliciting damage to either functional proteins, the mtDNA, or both (Sorensen et al., 2016). It has been established that an imbalance in the oxidant/antioxidant ratio stimulates atrophy pathways, impedes skeletal muscle growth and/or muscle turnover supressing the capacity to repair chemotherapy-induced damage (as reviewed in Sorensen et al., 2016). The net effect on the skeletal musculature would be wasting and a detrimental loss of force production capacity. While this has been increasingly established for the anthracycline chemotherapeutic, doxorubicin, which is a known pro-oxidant (Davies and Doroshow, 1986; Doroshow and Davies, 1986; Jones, 2006; Ashley and Poulton, 2009; Gilliam et al., 2012, 2013), and more recently established for combination colorectal cancer chemotherapy treatment regimens which include oxaliplatin (i.e., FOLFOX André et al., 2004; Aschele et al., 2005), there is currently no data describing the effects of OXA on the skeletal muscular system.

Here we investigate the experimental therapeutic BGP-15 (O-(3-piperidino-2-hydroxy-1-propyl)nicotinic amidoxime), which has previously been used in clinical trials for the treatment of skeletal muscle pathology associated with Type 2 Diabetes (through insulin sensitization; U.S. National Institutes of Health, 2014), Duchenne Muscular Dystrophy (DMD) and heart failure (through anti-inflammatory and anti-fibrotic mechanisms; Gehrig et al., 2012; Sapra et al., 2014). Via its action as a modulator of the cytoprotective response to cellular stress, and specifically as a poly (ADP-ribose) polymerase (PARP) inhibitor, heat shock protein-inducer (Sarszegi et al., 2012; U.S. National Institutes of Health, 2014), membrane lipid therapeutic (Salah et al., 2016) and an antioxidant inducer (Henstridge et al., 2014), BGP-15 has previously been shown to protect against skeletal muscle dysfunction, damage and wasting (Sapra et al., 2014; Kennedy et al., 2016; Salah et al., 2016). As such, it is of particular interest to us for protection against chemotherapy-induced skeletal muscle dysfunction due to its capacity to improve myopathic structural changes. Thus, we aimed to (1) investigate the effects of OXA treatment on skeletal muscle and mitochondrial function at the whole body, myocellular, and molecular level; and (2) evaluate the efficacy of BGP-15 co-treatment as a therapeutic avenue through which to protect the skeletal muscle during chemotherapy treatment. We hypothesized that OXA treatment would (1) induce skeletal muscle atrophy, wasting and/or pathology and (2) penetrate the mitochondria to induce mitochondrial pathology and dysfunction; and that (3) BGP-15 would preserve the skeletal muscle mass, as well as protect against OXA-induced perturbations in muscle morphology and mitochondrial health, thus reducing the impact of chemotherapy treatment on the skeletal muscular system.

## Materials and methods

### Ethics approval

All experimental procedures were approved by the Victoria University Animal Ethics Experimentation Committee and conformed to the Australian Code of Practice for the Care and Use of Animals for Scientific Purposes.

### Animals

Six-week old male Balb/C mice were obtained from the Australian Resource Centre (Western Australia, Australia) and were acclimatized for a minimum of 3 days before being randomly assigned to treatment groups (*n* = 8). Mice were housed in groups of 4–5 and maintained on a 12 h light/dark cycle with *ad-libitum* access to standard rodent chow and water. Prior to treatment (on day 1), mice were scanned for body composition. Mice were then treated with either vehicle (0.1% DMSO; VEH), OXA (3 mg/kg; Sigma Aldrich, Australia) or OXA with BGP-15 (15 mg/kg, kindly donated by N-gene R&D, Australia; OXAB) via intraperitoneal injection on days 1, 3, 5, 8, 10, and 12. The cumulative OXA dosage used in our study is equivalent to that given to humans, scaled for the metabolic activity of mice according to Reagan-Shaw et al. (2008). The concentrations and injection protocol for OXA have been used and published by our collaborators previously (Stojanovska et al., 2015; McQuade et al., 2016b), while the BGP-15 dosage administered has proven efficacious against murine myopathies (Chung et al., 2008; Gehrig et al., 2012; Kennedy et al., 2016). On day 14, mice were housed in the Promethion metabolic system for 24 h and on day 15, scanned again for changes in body composition, prior to being anaesthetized (sodium pentobarbitone, 60 mg/kg). When no reflexes were present, muscles and organs were harvested. Food and water consumption was monitored on treatment days.

### Body composition analysis

Directly prior to the first treatment on Day 1 and prior to non-recovery surgery on Day 15, mice were analyzed for body composition (lean, fat and water mass) using an echo magnetic resonance imaging (echoMRI) body composition analyser (EMR-150, Echo Medical Systems, USA). Scans were conducted in triplicate at each time point and included both the standard and water phases of analysis, with a 30-s time-lapse between each scan. Data is presented as the mean of the three scans. Composition results are expressed as an index against body weight (measure/body weight) with pre-treatment data not shown since no significant differences between groups were observed.

### Metabolism, voluntary exercise capacity, and behavioral analysis

Mice were housed individually in a Promethion metabolic cage system (Sable Systems, USA) for 24 h after the cessation (day 14–15) of treatment. Respiratory gases were measured with an integrated fuel cell oxygen (O_2_) analyser, spectrophotometric carbon dioxide (CO_2_) analyser and capacitive water vapor partial pressure analyser (GA3, Sable Systems, USA). Gas sampling was recorded at 1 s intervals with water vapor, pressure and temperature controlled for, to assess indirect calorimetry. Gas sensors were calibrated weekly with 100% N_2_ (zero reference for all other gas and vapor) and a span gas with known concentrations of O_2_ and CO_2_. The Promethion system utilizes a pull-mode, negative pressure system, through a multi-channel mass flow generator which measures and controls airflow (FR8, Sable Systems, USA) at an incurrent flow rate of 2,000 mL/min. Respiratory quotients (RQ) were calculated as a ratio of CO_2_ production to O_2_ consumption with energy expenditure calculated using the Weir equation: Kcal/hr = 60^*^(0.003941^*^VO_2_ + 0.001106^*^VCO_2_) (Kaiyala et al., 2012). Mouse ambulatory activity and position (x, y, and z axis, 0.25 cm spacing) within the cages was recorded continuously (BXYZ-R Sable Systems, USA). Mice also had free access to running wheels with revolutions recorded using a magnetic reed switch. Mice were permitted *ad libitum* access to food and water hoppers which were suspended from load cells to continuously record interaction with food and water, as well as consumption. Measures of time spent during behavioral activities were derived from the ethoSCAN behavioral macro supplied by Sable Systems (USA), with short lounges identified as periods of inactivity <60s and long lounges >60s. Time spent on particular activities were calculated as a percentage of total time spent in the cage (total 24 h).

### Histology

Following excision of tibialis anterior (TA) from mice, muscles were coated in OCT compound and snap frozen in liquid nitrogen-chilled isopentane (Sigma Aldrich, Australia). Frozen OCT embedded TA muscles were cryosectioned (10 μm), stained and mounted according to specific protocols as described below. Images of the whole section were captured using a Zeiss Axio Imager Z2 microscope (Carl Zeiss MicroImaging GmbH, Germany) at 10x magnification and a further magnification of 200x was used for greater detailed images.

#### Haematoxylin & eosin (H&E)

Slides were stained using a standard H&E staining protocol (30 s incubation in haematoxylin and 1 m 45 s incubation in eosin; Timpani et al., 2016) and mounted with DPx (BDH, Poole, UK). To determine fiber size frequency distributions, 200 fibers per section (except for those on the periphery that were cropped) were individually circled on three images taken from the top, middle and bottom along the midline from each TA cross section. ImageJ (NIH, USA) measurement analysis was used for measurement data. Since the TA has been shown to localize different fiber types to different areas of the muscle this approach was used to limit the influence of fiber type variations within the muscle cross section (Wang and Kernell, 2001; Shortreed et al., 2009).

#### Oil Red O (ORO)

Air dried TA sections were fixed in 3.7% formaldehyde for 60 m before being rinsed in x3 individual deionized water baths for 30 s each. Samples were then incubated in Oil Red O working solution [5:1 Oil Red O (Sigma Aldrich, Australia) in 60% triethyl-phosphate (w/v)] for 30 min. Thereafter, samples were washed three times in individual deionized water baths for 30 s each, then rinsed in running tap water for 10 m before being mounted with 10% glycerol in PBS. Images were converted to 8bit photos and thresholded, then analyzed by assessing the intensity of black to white. The full cross section of the TA was imaged and data is expressed as the Oil Red O positive area (black) as a percentage of the total cross sectional area (black + white).

#### Alizarin red

The Ca^2+^ content of TA sections was assessed using Alizarin Red (TMS-008-C, Merck Millipore), a dye which chelates with calcium to form Alizarin Red S-Ca^2+^ complexes. Slides were stained for 2 m with Alizarin Red, dipped in 100% acetone 20 times, then dipped in 1:1 acetone-xylene 20 times, before being washed in 100% xylene for 1 m. Samples were mounted with DPx then the whole cross section of the TA was photographed. Images were converted to 8bit photos then analyzed by assessing the intensity of black of the total cross-sectional area. Data is expressed as arbitrary units.

#### Gomori trichrome

Gomori Trichrome (LG) stain (HT10316, Sigma Aldrich, Australia) distinguishes three muscle components: (1) muscle (red/pink), (2) nuclei (blue/black), and (3) collagen (green-blue). To achieve this stain, samples were bathed in hematoxylin for 1 m before being washed in tap water until the water ran clear. Samples were then stained with Gomori trichrome for 30 s and dipped in tap water 20–25 times before being dipped in a 0.2% acetic acid bath 20 times, then bathed in 0.2% acetic acid for 30 s. Samples were then bathed for 30 s increments in x4 baths containing 95% (x2) and 100% (x2) ethanol, before being bathed in xylene for 1 m. Thereafter, samples were mounted with DPx. The intensity of red (muscle), blue (nuclei) and green (collagen) pixels were assessed using ImageJ. The full cross section of the TA was imaged and data is expressed as the percentage of collagen (green) within the total cross sectional area.

#### Succinate dehydrogenase (SDH)

SDH is an enzyme located in the mitochondria that oxidizes succinate to fumarate. This reaction, in the presence of nitro blue tetrazolium, is demonstrated by the formation of a blue-purple product with more intensely colored fibers indicating highly oxidative fibers with a greater mitochondrial density. Slides were incubated in working solution (0.2M sodium succinate, 0.2M PBS, 0.05% nitro blue tetrazolium, pH 7.6) for 60 min at 37°C, fixed in formal saline (0.9% NaCl, 10% formaldehyde) and mounted with glycerol jelly. Images were converted to 8bit, thresholded and SDH intensity was measured in full cross sections of the TA.

### Platinum detection in subcellular fractions

#### Subcellular fractionation

TA muscles were homogenized in buffer (containing: 100 mM potassium chloride, 50 mM tris (hydroxymethyl)aminomethane, 5 mM magnesium chloride hexahydrate, 1.8 mM adenosine triphosphate, 0.5 mM ethylenediaminetetraacetic acid; pH 7.2). Tissue homogenates were transferred to Eppendorf tubes and centrifuged at 650G for 3 min at 4°C. The supernatant, which contains the mitochondria, was decanted into separate Eppendorf tubes. The pellet which contains the nuclear fraction was resuspended in RIPA lysis buffer (25 mM tris(hydroxymethyl)aminomethane hydrochloride, 150 mM sodium chloride, 1% sodium deoxycholate and 0.1% sodium dodecyl sulfate; pH 7.6) and further diluted to a total volume of 4 mL in MilliQ water. The mitochondrial sample was centrifuged at 15,000G for 3 min at 4°C. The supernatant was discarded and the mitochondrial pellet was resuspended in 4 mL of MilliQ water.

#### Atomic absorption spectrophotometry

Once the nuclear and mitochondrial fractions were derived, samples were aspirated into a Shimadzu AA-6300 Atomic Absorption Spectrophotometer (AAS). The specific AAS conditions used to carry out these analyses included an air-acetylene flame, with a fuel flow of 1.5 L/min and an air flow of 15 L/min. The burner height was optimized for each element. Due to the analytical wavelength used (265.9 nm for Pt), background correction was required—this was supplied by a D_2_ lamp using a slit width of 0.7 nm and a current of 25 mA (Pt). Samples were aspirated, with three repeat measurements recorded following an initial 2 s pre-spray time. Individual measurements were taken by averaging the absorbance readings over 3 s, which also allowed the calculation of a relative mean square percentage (RMS%) uncertainty. These three measurements were then averaged to give a final absorbance reading for each sample. Standard calibration curves were also produced before each daily run of samples, with concentration ranges of 10-40 ppm Pt utilized. Concentration values for the unknown samples were calculated automatically by the Shimadzu AAWizard software.

### Mitochondrial viability and mtROS

#### Isolation of flexor digitorum brevis (FDB) fibers

FDB fibers were isolated according to procedures described by Schuh et al. (2012). Following surgical excision of the FDB from both feet, the muscles were incubated in 1 mL of pre-warmed dissociation media (DMEM, Gibco 10566016; 4.5 mg/ml glucose, 2% FBS, Bovogen Biologicals; 4 mg/mL collagenase A, Roche 10103586001; 50 μg/mL gentamycin, Sigma Aldrich, Australia G1397) for 1 h 45 min (37°C, 5% CO2). Following the incubation period, FDB muscles were removed from collagenase and placed into ~1.5 mL of incubation media [DMEM containing 4.5 mg/ml glucose, no phenol red (Gibco), 2.0% FBS, 0.1% Gentamycin solution (Sigma Aldrich, Australia G1397)] and triturated with pipette tips of decreasing bore size to yield isolated fibers. Fibers were then plated according to analysis methods described below.

#### Determination of mitochondrial viability

Mitochondrial viability was assessed using the fluorescent probes MitoTracker Green and Red (Molecular Probes, Australia). MitoTracker Green is a non-selective mitochondrial dye that labels all mitochondria irrespective of the mitochondrial membrane potential, while MitoTracker Red only permeates the mitochondrial matrix and fluoresces in the presence of an inner mitochondrial membrane potential (ΔΨ). Mitochondrial viability was calculated as the percentage ratio of active mitochondria (MitoTracker Red) to the total mitochondrial pool (MitoTracker Green).

Fifty microliters of isolated FDB fibers suspended in incubation media [DMEM containing 4.5 mg/ml glucose, no phenol red (Gibco), 2.0% FBS, 0.1% Gentamycin solution (Sigma Aldrich, Australia G1397)] was plated onto matrigel (Sigma Aldrich, Australia, E1270) coated 96 well microplates (all samples were run in triplicate) and confluency was determined using a light microscope. If ~60% of the well bottoms was not covered by isolated FDB fibers, an additional 50 μL aliquot of fibers was dispensed into the well. For wells that did not receive the additional 50 μL of fibers, 50 μL of incubation media was added and the microplate was incubated overnight.

Ten minutes prior to the addition of the MitoTracker dyes, FCCP and antimycin A (final concentration of 3 μM each, Sigma Aldrich, Australia) were added to the positive control wells. As FCCP induces the collapse of the mitochondrial membrane potential and antimycin A inhibits complex III function, mitochondrial viability decreases as evidenced by a reduction in the intensity of MitoTracker Red fluorescence. Following this, a cocktail of MitoTracker Green and Red (final concentration of 200 and 50 nM, respectively. Molecular Probes) in Flurobrite media (50 μL total, ThermoFisher, Australia) was added to each well and incubated at 37°C for 3 min. Fibers were then washed twice with Flurobrite media and imaged on an Olympus Inverted Fluorescence Microscope (IX-81, Olympus, Tokyo, Japan) using FITC and TRITC filters and a standardized exposure time. Three random images were taken of each well in a blinded fashion and with standardized exposure and brightness settings, and the average intensity of red and green fluorescence of each image analyzed using ImageJ. Relative MitoTracker Red fluorescence as a proportion of relative MitoTracker Green fluorescence was calculated to give a value of mitochondrial viability percentage. The RFU MitoTracker Green stain were also expressed as arbitrary units of mitochondrial density/population. Analyzed images were all taken using standardized imaging settings, such as ISO and exposure times.

#### Determination of mitochondrial superoxide production

Isolated fibers suspended in incubation media were prepared as mentioned in the previous section. Fibers were labeled with MitoSOX Red mitochondrial superoxide indicator (Molecular Probes) to detect superoxide production. MitoSOX reagent stock solution (5 mM) was diluted in a HBSS/Ca/Mg buffer (10 mM HEPES, 150 mM NaCl, 5 mM KCl, 1 mM MgCl_2_ and 1.8 mM CaCl_2_, pH 7.4) at 37°C and added to the fibers. Fibers were incubated at 37°C in 5% CO_2_ for 3 min. Following this the staining solution was removed and, as a counterstain, cells were labeled with MitoTracker Green for 30 min. After counterstaining, cells were washed twice with incubation media and live cells were photographed using an IX-81 Olympus Inverted Fluorescence Microscope. Images were quantified using ImageJ software. Data is expressed as MitoSOX Red RFU relative to the total mitochondrial pool (MitoTracker Green RFU). Three images were taken of each well in a blinded fashion and the average intensity of red and green fluorescence of each image was analyzed using ImageJ. Analyzed images were all taken using standardized imaging settings, such as ISO and exposure times.

### Muscle protein extraction and western blotting

Frozen TA muscles were mechanically disrupted (TissueLyser, Qiagen, Germany) for 30 s at 30 Hz in homogenizing buffer (0.125 M Tris-HCl, 4% SDS, 10% Glycerol, 10 mM EGTA, 0.1 M DTT) containing 0.1 μL.mL^−1^ of protease and phosphatase inhibitor cocktail (Sigma Aldrich, Australia), and vortexed, followed by a freeze-thaw cycle. Protein concentration was then determined using a commercially available assay (Red 660, G-Biosciences, Astral Scientific, Gymea, Australia), and samples were diluted to equivalent concentrations (1 μg.μL^−1^) in homogenizing buffer. Bromophenol blue (1% v/v) was then added before heating to 95°C for 5 min. Samples were loaded into pre-cast 26 well stain-free 8–16% gradient gels (Criterion™ TGX Stain-Free™ Precast, BioRad, Gladesville, Australia) at a concentration of ~8 μg protein per lane with all constituents present (i.e., no centrifugation). Molecular weight marker (PageRuler® Plus, Thermo Scientific, Australia) was loaded, along with five lanes of increasing volume of a pooled-sample to generate five-point calibration standard curves on each gel. This was used for quantification of sample blot intensities relative to the standard curve both within and between gels, and ensures sample blot intensities are within the linear range of the signal (i.e., primary antibody binding is not saturated; Murphy and Lamb, 2013). After separation by SDS PAGE, stain-free gels were activated by UV light (ChemiDoc™ MP, BioRad, Gladesville NSW, Australia) and imaged to visualize the total protein of each lane before the proteins were transferred to PVDF membranes (Trans-Blot® Turbo™, BioRad, Gladesville NSW, Australia). Membranes were then blocked in 20 mM Tris, 150 mM NaCl, and 0.1% Tween 20 (TBST) containing 5% w/v non-fat milk powder for 1 h at room temperature, then washed in TBST. After this, membranes were incubated overnight at 4°C with rocking, using the following primary antibodies diluted 1:1,000 in TBST containing 5% w/v BSA and 0.1% w/v sodium azide. Probes used: Bax total (CST #2772), MuRF1 total (ECM #MP3401), PAR total (Enzo #ALX-804-220), PARP-1 total (Santa Cruz SC-8007), PARP-2 total (Santa Cruz SC-393310), p70S6K total (CST #2708), rp-S6 total (C ST #2217), SQSTM1/p62 total (CST #5114). Membranes were subsequently washed with TBST, then probed with appropriate horseradish peroxidase-conjugated secondary antibody (PerkinElmer, Australia) diluted 1:50,000 in 5% non-fat milk TBST for 1 h at room temperature. Protein-antibody-HRP conjugates were visualized using super sensitive ECL detection (SuperSignal® West Femto, Thermo Scientific, Australia), imaged (ChemiDoc™ MP, BioRad, Australia), then analyzed using software (ImageLab v5.1, BioRad, Australia). Total protein loading was determined from stain-free gel images to measure intensity of each lane, which was expressed relative to the linear regression of the standard curve (Ashley and Poulton, 2009). These values were then used as a loading control to normalize all blot values for proteins of interest. The proteins selected were done so to cover a broad spectrum of cellular degradation pathways, including atrophy, autophagy, protein synthesis, to preliminarily investigate how OXA induces lean muscle mass loss. Further, PARP was assessed due to BGP-15's inhibitory effect on PARylation.

### Statistical analysis

Statistical analysis was performed using Graphpad Prism 7 software using one-way analysis of variance to detect treatment effects and Tukey post hoc tests for multiple group comparison. An α value of 0.05 was considered statistically significant. Data is presented as means ± SEM.

## Results

### Body weight and food consumption

**Figure 1:**
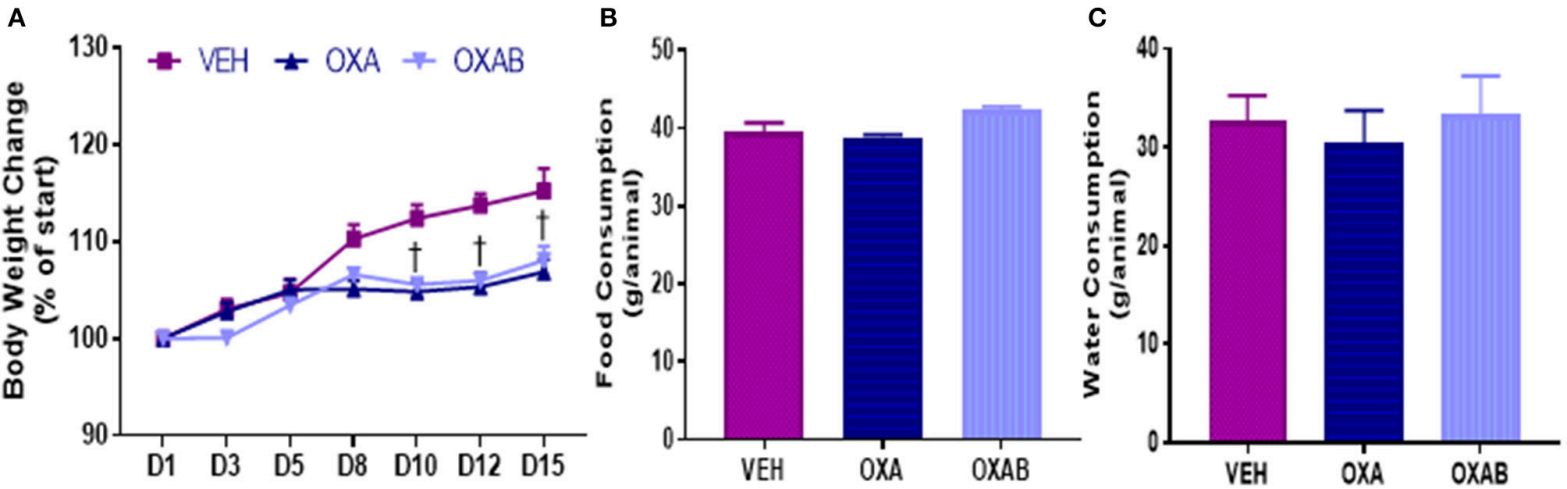
**Body weight and food consumption over the treatment period**. OXA significantly reduced final post treatment body weights, with weight loss plateauing from D8 of OXA treatment **(A)**. Body weight of OXA treated mice (including OXAB) was significantly reduced from VEH from D10 onwards. No differences were detected in **(B)** Food consumption or **(C)** Water consumption between the groups. D#, treatment day number; Significance, ^†^*p* < 0.005 OXA and OXAB different from VEH. *n* = 6–8.

Reduced food consumption due to gastrointestinal side-effects (McQuade et al., 2016b) and nausea (Love et al., 1989; Greene et al., 1993) is associated with chemotherapy treatment in humans, and as such, body weight and food and water intake were monitored. From days 1 through 8 of treatment, there were no significant differences in body weight gain observed between groups (*p* > 0.05, Figure 1A), however, weight gain in OXA and OXAB treated mice plateaued at day 8 and remained significantly lower than VEH from D10 onwards (*p* < 0.005; Figure 1A). There were no significant differences between the groups in food (Figure 1B) or water (Figure 1C) consumption over the treatment period (*p* > 0.05).

### BGP-15 protects against OXA induced-lean and fat mass loss but increases heart/body weight ratio

**Figure 2:**
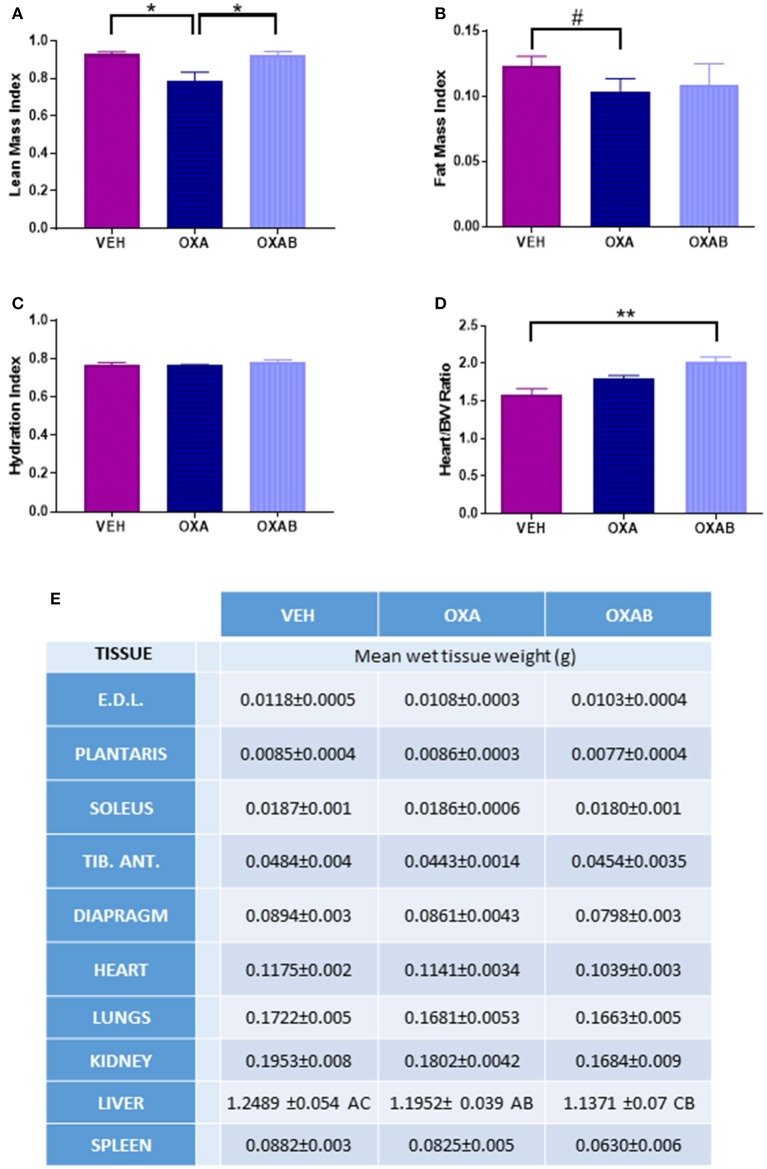
**BGP-15 protects against Oxaliplatin-induced lean tissue but not fat mass loss. (A)** BGP-15 protected against the OXA-induced reduction in LMI. **(B)** FMI was reduced by OXA treatment by 15% with no protection afforded by BGP-15. **(C)** Hydration Index calculated by [(echo derived total water – echo derived free water)/echo derived lean mass]. **(D)** Heart weight indexed against body weight showed OXAB treatment significantly increased heart size in relation to body weight. **(E)** Absolute wet tissue weights showed no significant difference either as raw or indexed against body weight, however OXA treatment reduced liver size with adjunct BGP-15 therapy further exacerbating this reduction (Table significance: A = *p* < 0.05 OXA to VEH. B = *p* < 0.05 OXA to OXAB. C = *p* < 0.0001 OXAB to VEH). BW, Body weight; EDL, Extensor Digitorum Longus; TID. ANT, Tibialis Anterior. Significance: ^*^p < 0.05, ^**^p<0.005; Trend: ^#^*p* = 0.076. *n* = 6–8.

The effects of OXA treatment on body composition, and the capacity for BGP-15 to protect against the loss of lean tissue was investigated in mice using MRI. OXA treatment induced a 15% reduction in the lean mass index (*p* < 0.05; Figure 2A) with this loss being completely protected against via adjunct treatment with BGP-15 (*p* < 0.05). The loss of lean tissue observed was not associated with changes in hydration status (*p* > 0.05, Figure 2C), however there was a trend for OXA treatment to reduce the fat mass index by 15% (*p* = 0.075; Figure 2B) with BGP-15 treatment affording no protection against this measure (*p* > 0.05, Figure 2B).

The wet weight of individual hind limb skeletal muscles, organs and the diaphragm was measured immediately after excision. There were no significant changes in wet tissue weights between treatment groups, with the exception of the liver which was reduced by 5% following OXA treatment (*p* < 0.05 from VEH, Figure 2E) and a further 5% following OXAB treatment (*p* < 0.0001 from VEH, Figure 2E). Although, there was no effect of treatments on the absolute heart weight, increased heart size when normalized to body weight was observed between OXAB and vehicle (*p* < 0.005, Figure 2D), however, no significant differences were observed for other collected muscles and organs when indexed against body weight.

### Metabolic and exercise capacity is unaffected by OXA treatment

**Figure 3:**
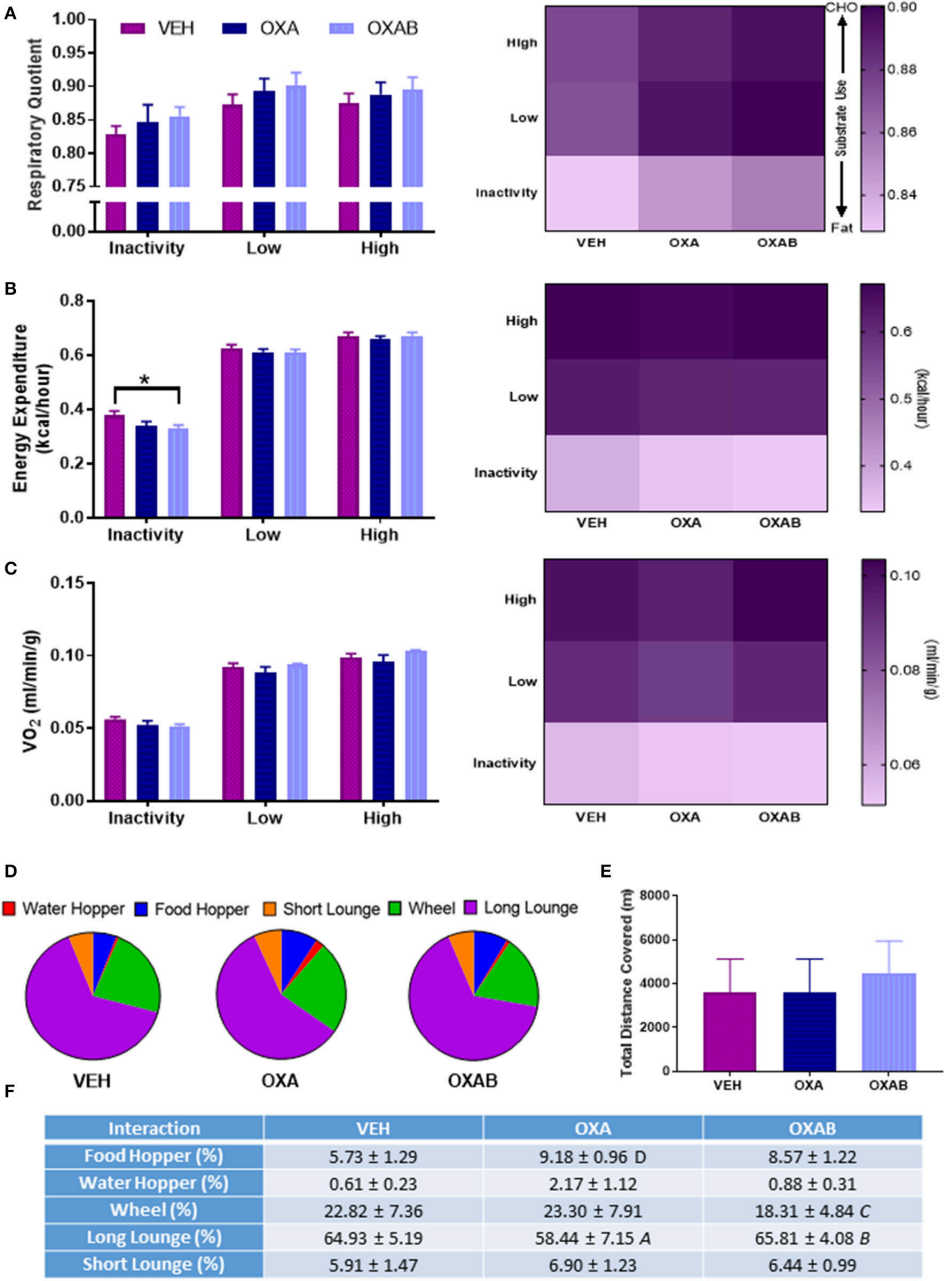
**BGP-15 reduces basal energy expenditure with OXA treatment not affecting exercise capacity. (A)** There was no significant effect of treatment on the respiratory quotient. **(B)** Energy expenditure at rest was reduced by BGP-15 adjunct therapy. **(C)** Oxygen consumption was not effected by treatment. **(D,F)** Time budgets of activities of daily living: OXA treatment reduced time spent engaged in long lounges (*p* < 0.05) while OXAB treatment reduced the time spent engaged in voluntary wheel running. **(E)** There was no effect of treatment on total meters covered (combination of wheel and pedestrian meters). CHO, Carbohydrate; VO_2_, Volume Oxygen. Table significance: **(A)** *p* < 0.05 OXA vs. VEH, **(B)** *p* < 0.05 OXA vs. OXAB, **(C)** trend (*p* = 0.088) OXA vs. OXAB, **(D)** trend (*p* = 0.057) OXA vs. VEH. Significance: ^*^p < 0.05. *n* = 6–8.

To assess the impact of the effects of OXA on the skeletal muscular system at the whole body level, the metabolism, voluntary exercise capacity and participation in activities of daily living (behavioral time budgets) of mice were quantified in Promethion Metabolic cages (Sable Systems, U.S) for 24 h following the conclusion of the treatment regimen. Interestingly, no changes were noted in energy substrate utilization (Figure 3A), overall energy expenditure (Figure 3B), overall O_2_ consumption (Figure 3C) or in exercise capacity (Figures 3D–F), following OXA treatment. Of note however, OXAB treatment reduced the basal energy expenditure at rest (*p* < 0.05, Figure 3B) and had a tendency (*p* = 0.088) to reduce the overall time spent on the running wheel, albeit the same distance was covered when compared to other groups. Furthermore, OXA treatment significantly reduced the time spent in inactivity periods longer that 60 s (long lounges), suggesting that mice were spending less time resting (Figures 3D,F). Interestingly though, OXA treated mice spent more time interacting with the food hoppers as seen in Figure 3D.

### OXA treatment changes the fiber distribution of TA sections and reduces isolated FDB fiber diameter

**Figure 4:**
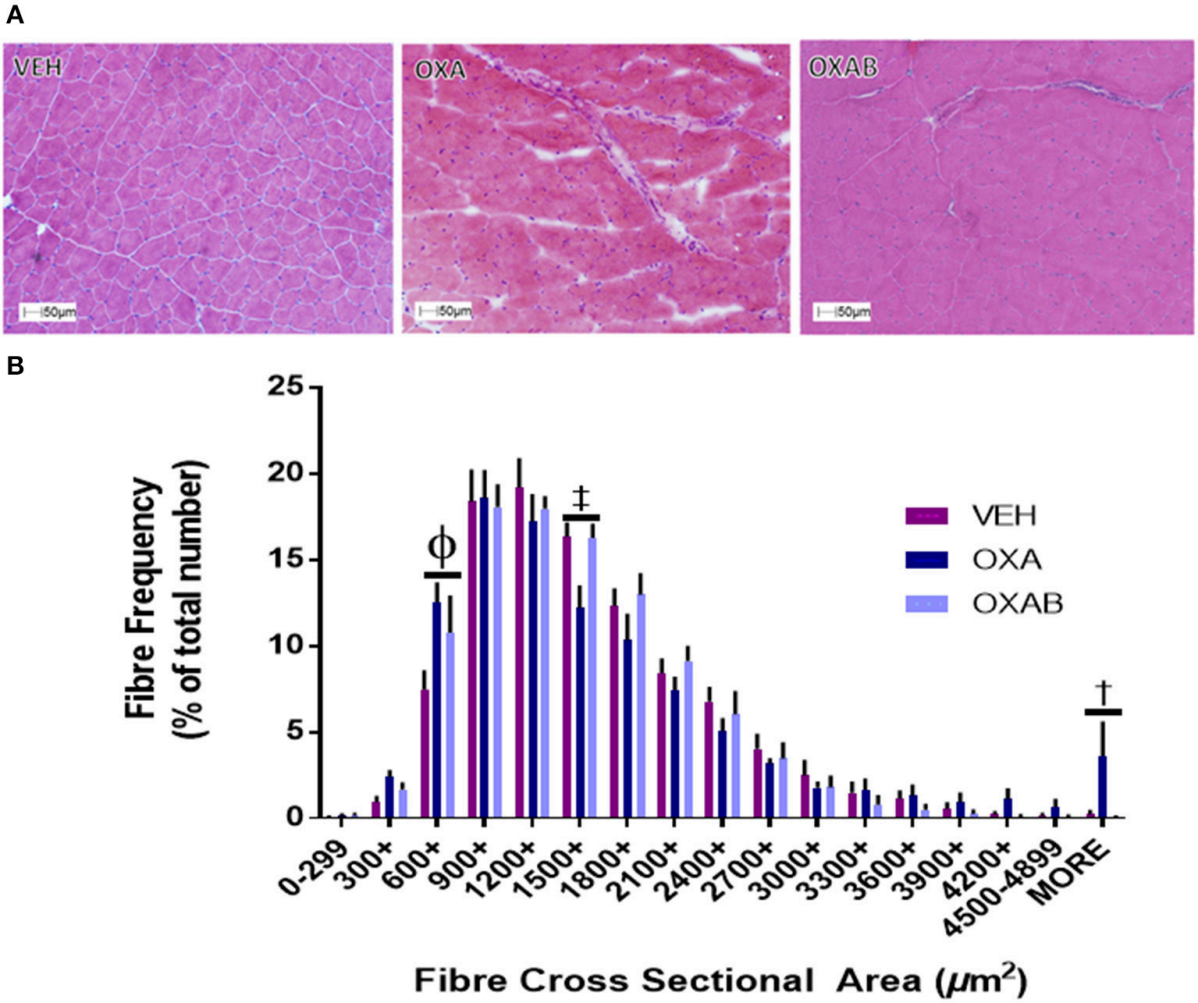
**OXA and BGP-15 treatment effect fiber size distribution in TA muscle**. **(A)** Representative images of H&E stained TA and fiber size frequency histogram **(B)** OXA treatment significantly increased the frequency of fibers in the 600-899 μm^2^ and >4,900 μm^2^ size bins whilst reducing the frequency of fibers in the 1,500–1,799 μm^2^ size bin. Significance: ^Φ^*p* < 0.0005 VEH compared to OXA and *p* < 0.05 VEH from OXAB; ^‡^*p* < 0.005 VEH and OXAB compared to OXA, ^†^*p* < 0.05 OXA and OXAB compared to VEH. *n* = 4.

With previous studies showing that the oxidative stress induced by some chemotherapeutic agents potentiates skeletal muscle dysfunction and atrophy (Bonifati et al., 2000; Gilliam et al., 2009), we were interested in assessing whether OXA could induce similar effects at the fiber level. OXA treatment increased the frequency of small TA fibers by 68% in the 600–899 μm bin range (*p* < 0.05), whilst reducing fiber frequency within the 1,500–1,799 μm bin range (*p* < 0.005). BGP-15 adjunct therapy offered protection exclusively against the OXA-induced fiber frequency reduction in the 1,500–1,799 μm range (Figure 4B). Myopathological analysis of H&E stained TA sections revealed no ultrastructural changes in nuclei location from the fiber periphery (normal) to the fiber center (pathological) and no signs of inflammatory infiltrate, which would both be indicative of damage and repair pathway activation (Figure 4A). In isolated FDB fibers, OXA treatment induced a 25% reduction in FDB fiber diameter (*p* < 0.0001 from VEH; **Figure 8F**). This effect was completely protected against by OXAB treatment (*p* < 0.0005 from OXA), whereby fiber diameter was comparable to VEH.

### BGP-15 protects against OXA-induced accumulation of intracellular Ca^2+^, fat and collagen in TA muscle

**Figure 5:**
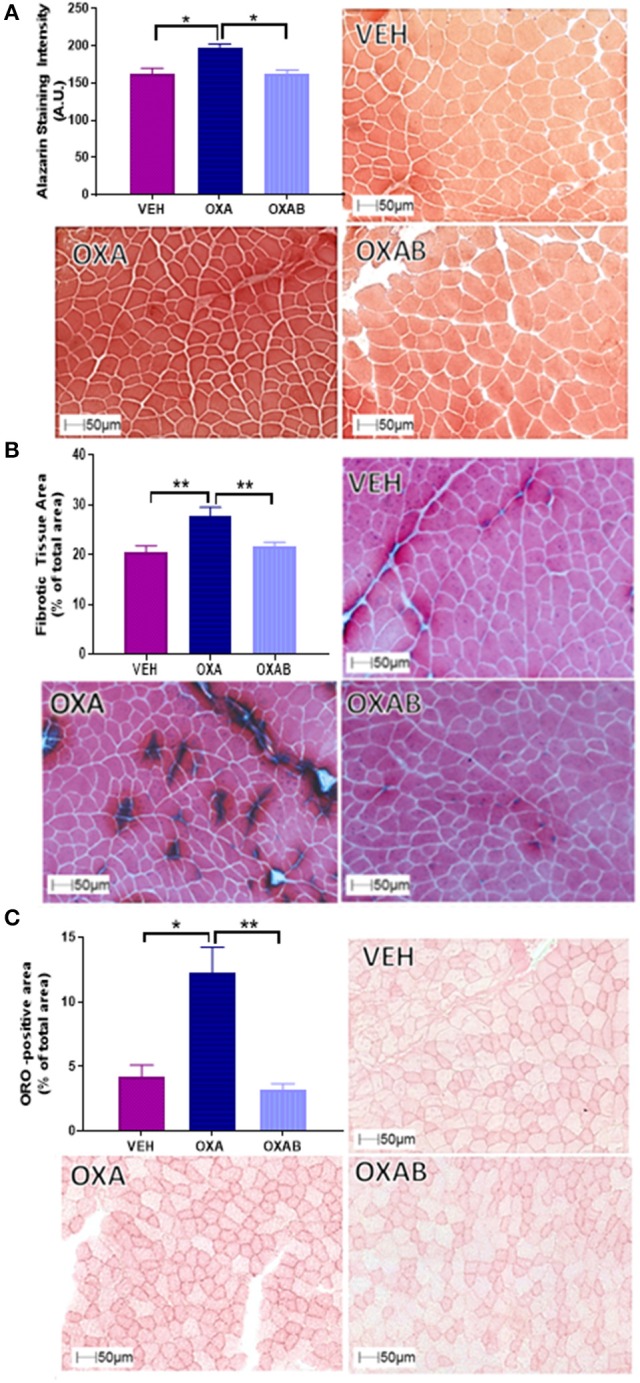
**BGP-15 protects against OXA-induced increases in intracellular Ca^**2+**^, collagen deposition and fat infiltration in TA muscle**. OXA treatment increased **(A)** intracellular Ca^2+^ content **(B)** fibrotic tissue and collagen deposition and **(C)** fat infiltration with OXAB protecting against all these parameters in TA muscles. A.U., Arbitrary units; ORO, Oil Red O. Significance: ^*^p < 0.05, ^**^p < 0.005.

Since intracellular Ca^2+^ dysregulation and accumulation is strongly linked with skeletal muscle pathology (Powers et al., 2007), we assessed the effect of OXA treatment on the Ca^2+^ content of TA using Alzarin Red staining (Figure 5A). OXA treatment increased the intracellular Ca^2+^ content by 22% from VEH (*p* < 0.05). This increase in Ca^2+^ however, could not be localized to a specific intracellular region (a limitation of the method) and did not appear to be at a concentration high enough to activate calpain-mediated damage responses as centralized nuclei were not observed in H&E stains (Figure 4A). Since fibrotic connective tissue and fat infiltration are also features of myopathy (Scheede-Bergdahl and Jagoe, 2013; Bredahl et al., 2016), Gomori trichrome and ORO staining were used to assess these markers, respectively. OXA treatment increased collagen deposition within TA sections by 36% (*p* < 0.0005, Figure 5B) and induced a 164% increase in the cross-sectional TA area infiltrated with neutral lipids (*p* < 0.05; Figure 5C). All OXA-induced histopathological features were completely protected against by OXAB treatment (Figures 5A–C).

### Mitochondrial Pt accumulation and increased SDH following OXA treatment

**Figure 6:**
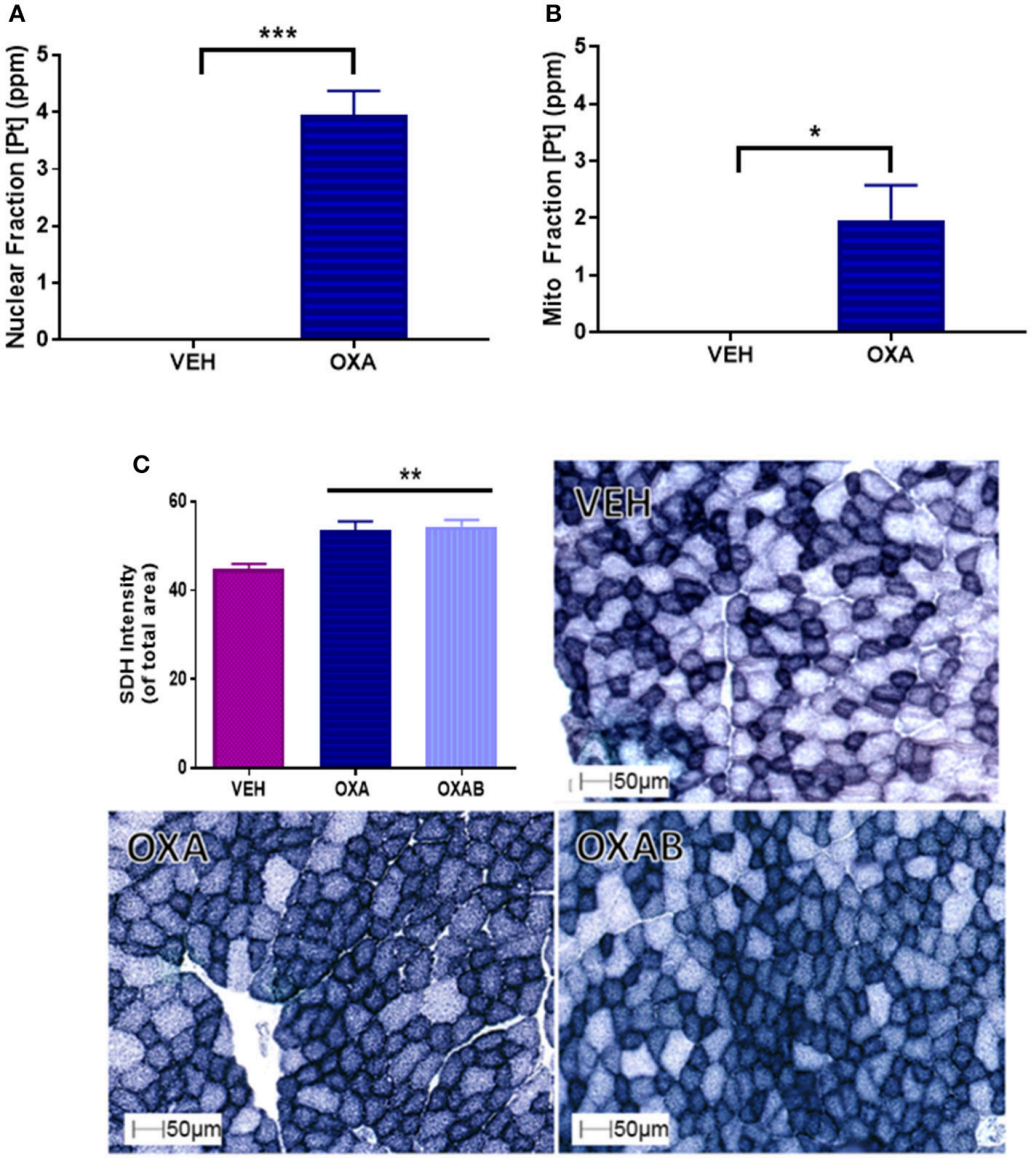
**OXA penetrates the nuclear and mitochondrial fractions of TA muscle homogenates and increases succinate dehydrogenase (SDH) content. (A)** OXA penetrated the nuclear and **(B)** mitochondrial fractions as quantified by Pt detection (*n* = 3–4). **(C)** SDH staining was significantly increased with OXA treatment but OXAB had no effect on this measure (*n* = 6–8). [Pt], Platinum concentration; SDH, SDH Succinate dehydrogenase; ppm, parts per million. Significance: ^*^p = <0.05, ^**^p = <0.005, ^***^p = <0.0005.

To establish whether OXA could (a) penetrate the skeletal muscle and (b) penetrate the double membrane of the mitochondria; analysis of the skeletal muscle nuclear and mitochondrial fraction was performed. We have previously hypothesized that OXA accumulation within the mitochondria could be deleterious to mitochondrial function (Sorensen et al., 2016). Pt was detected in both the nuclear (3.95 ppm) and mitochondrial (1.97 ppm) fractions compared to VEH (*p* < 0.05, Figures 6A,B, respectively). Of interest, there was ~50% less Pt content within the mitochondria subcellular fraction than in the nuclear fraction. To assess the impact of OXA permeation on mitochondrial capacity, we next investigated the content of the mitochondrial enzyme, SDH in TA sections (Figure 6C). There was a significant increase (~20%) in the SDH content of OXA treated mice compared to VEH (*p* < 0.005; Figure 6C), however BGP-15 had no effect on this parameter.

### OXA treatment does not induce PARP expression in skeletal muscle

PARP activation occurs secondary to DNA damage, and as such, we next investigated whether OXA treatment could induce PARP. Of particular interest was whether the PARP-inhibitor BGP-15 could inhibit induction of PARP activity. As such we quantified PARP1, PARP2, and total PARylation (marker of PARP activity, Gibson and Kraus, 2012, Figure 7) in TA muscles. Surprisingly there was no effect of OXA treatment on either PARP1 or 2 expression or total PARylation in TA. To this extent, there was no effect of BGP-15 on these parameters since PARP was not activated.

**Figure 7:**
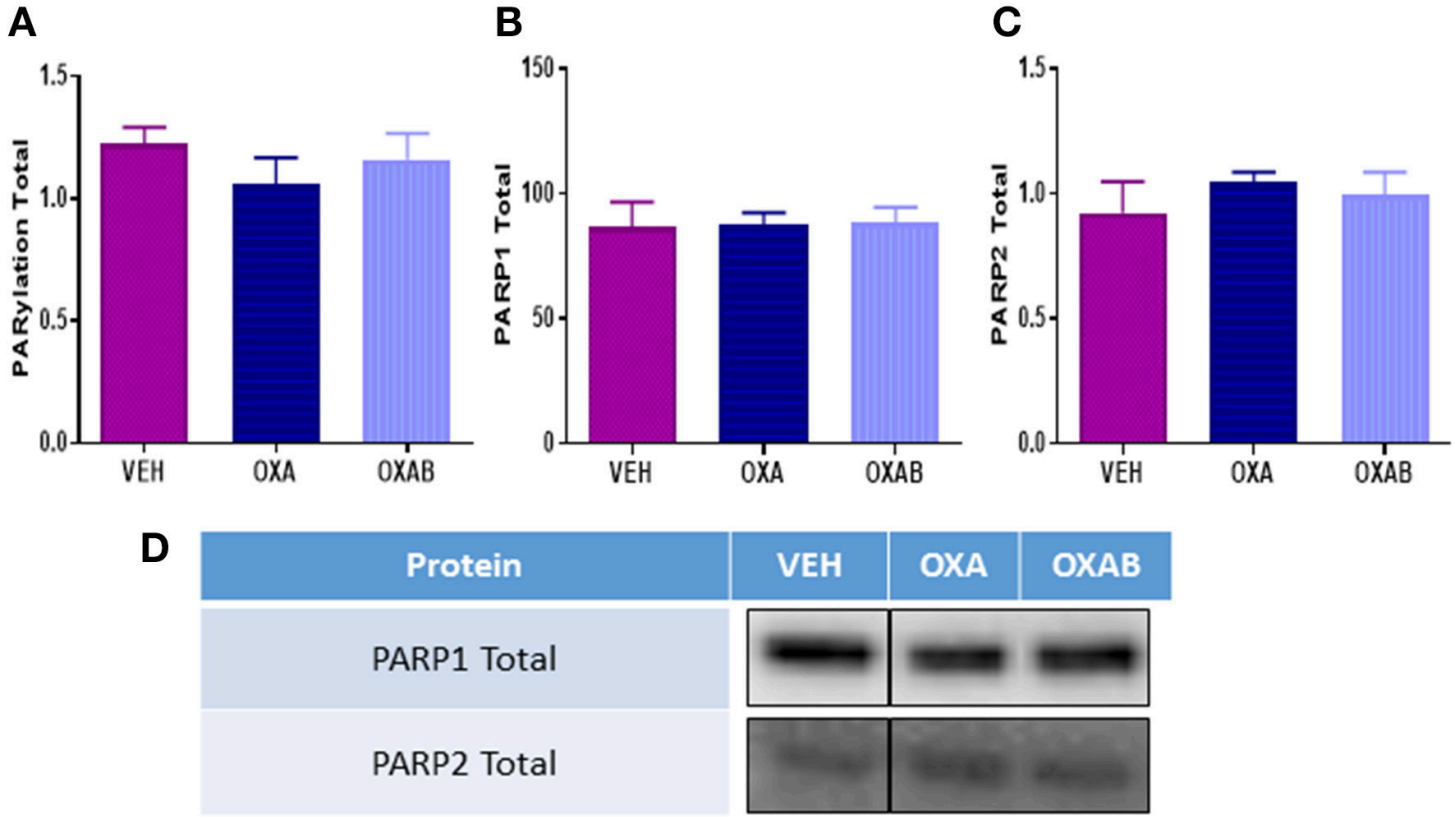
**OXA and BGP-15 treatment does not alter PARP expression in mouse TA**. No changes in **(A)** total PARylation **(B)** PARP1 or **(C)** PARP2 were detected following OXA treatment. Adjunct treatment with the PARP inhibitor BGP-15 (OXAB) did not alter PARP expression. **(D)** Representative stainfree Western blot images are digitally cut together to remove blots of other chemotherapies not presented in this manuscript, no other alteration was performed.

### BGP-15 adjunct treatment improves mitochondrial viability and protects against OXA-induced mitochondrial changes

**Figure 8:**
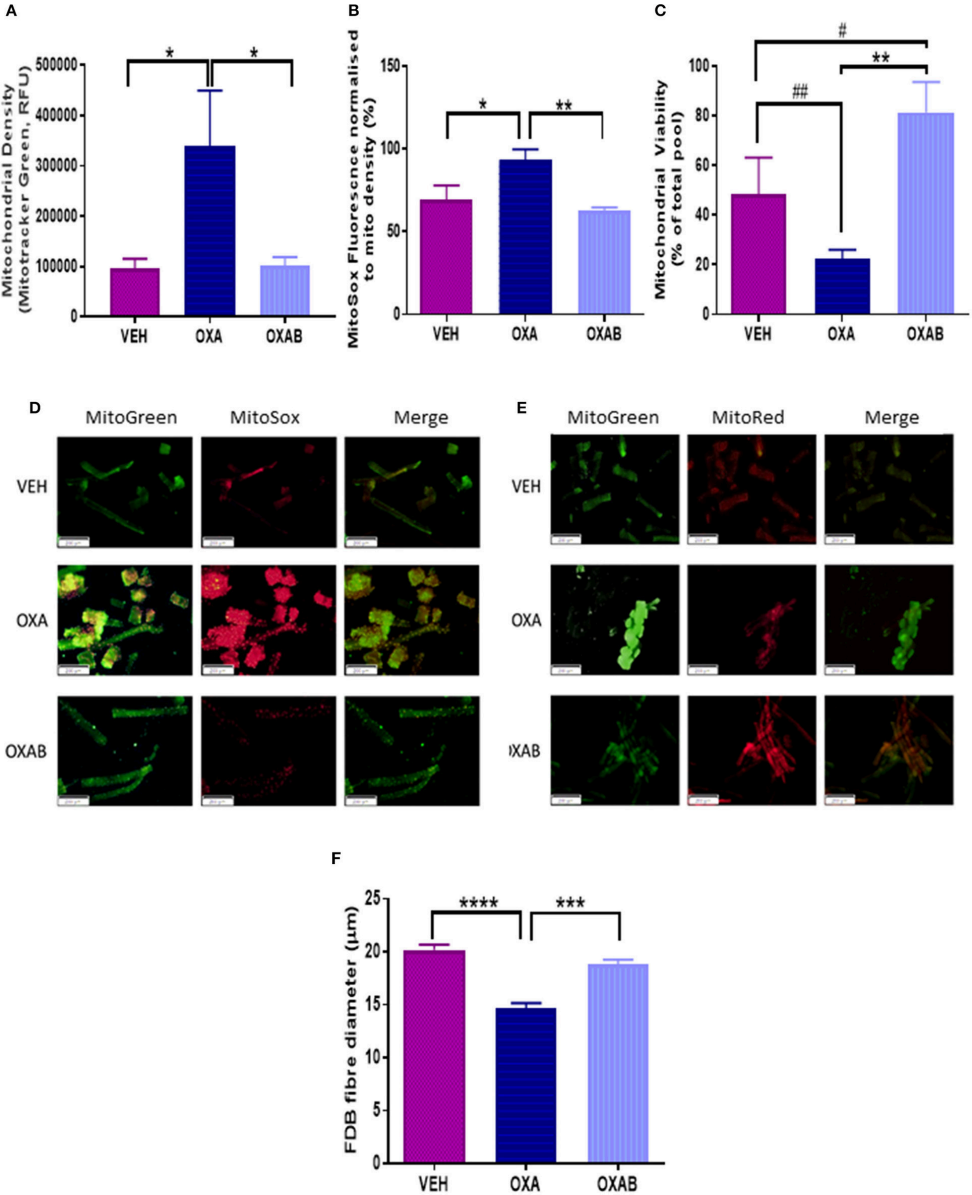
**BGP-15 protects against OXA-induced mitochondrial superoxide production and fiber diameter loss and improves mitochondrial viability in FDB fibers**. OXA treatment induced **(A)** A 4-fold increase in mitochondrial density, with OXAB protecting against this increase. **(B)** OXAB protected against a 25% increase in MitoSOX fluorescence induced by OXA treatment. **(C)** OXA treatment reduced mitochondrial viability while OXAB treatment protected against the effects of OXA and improved viability 4-fold from VEH. Representative images of MitoSOX and MitoTracker stained FDB fibers **(D,E)**. OXA treatment also induced a **(F)** 25% reduction in FDB Fiber diameter with OXAB protecting against this reduction. FDB, Flexor Digitorum Longus; Mito, Mitochondria; SOX, Superoxide. Significance: ^*^p < 0.05, ^**^p < 0.005, ^***^p < 0.0005, ^****^p < 0.0001. Trend: ^#^*p* = 0.073, ^*##*^*p* = 0.077. *n* = 4–6.

mtROS are prevalent mediators of various muscle wasting mechanisms, including atrophy and apoptosis, and their production can be exacerbated if the mitochondria become dysfunctional or damaged (Kirkinezos and Moraes, 2001; Lenaz et al., 2002; Kujoth et al., 2005; Le Bras et al., 2005; Holzerová and Prokisch, 2015). At the mitochondrial level, OXA treatment induced a significant increase in mitochondrial density (*p* < 0.05, Figure 8A) and superoxide production (*p* < 0.05, Figure 8B) in FDB fibers, with OXAB treatment protecting against these effects. Interestingly, BGP-15 significantly increased mitochondrial viability 4-fold from OXA treated fibers (*p* < 0.005, Figure 8C) and 2-fold from VEH (*p* = 0.073) with OXA treatment alone showing a trend to decrease viability from VEH levels (*p* = 0.077).

### OXA reduces protein synthesis markers

**Figure 9:**
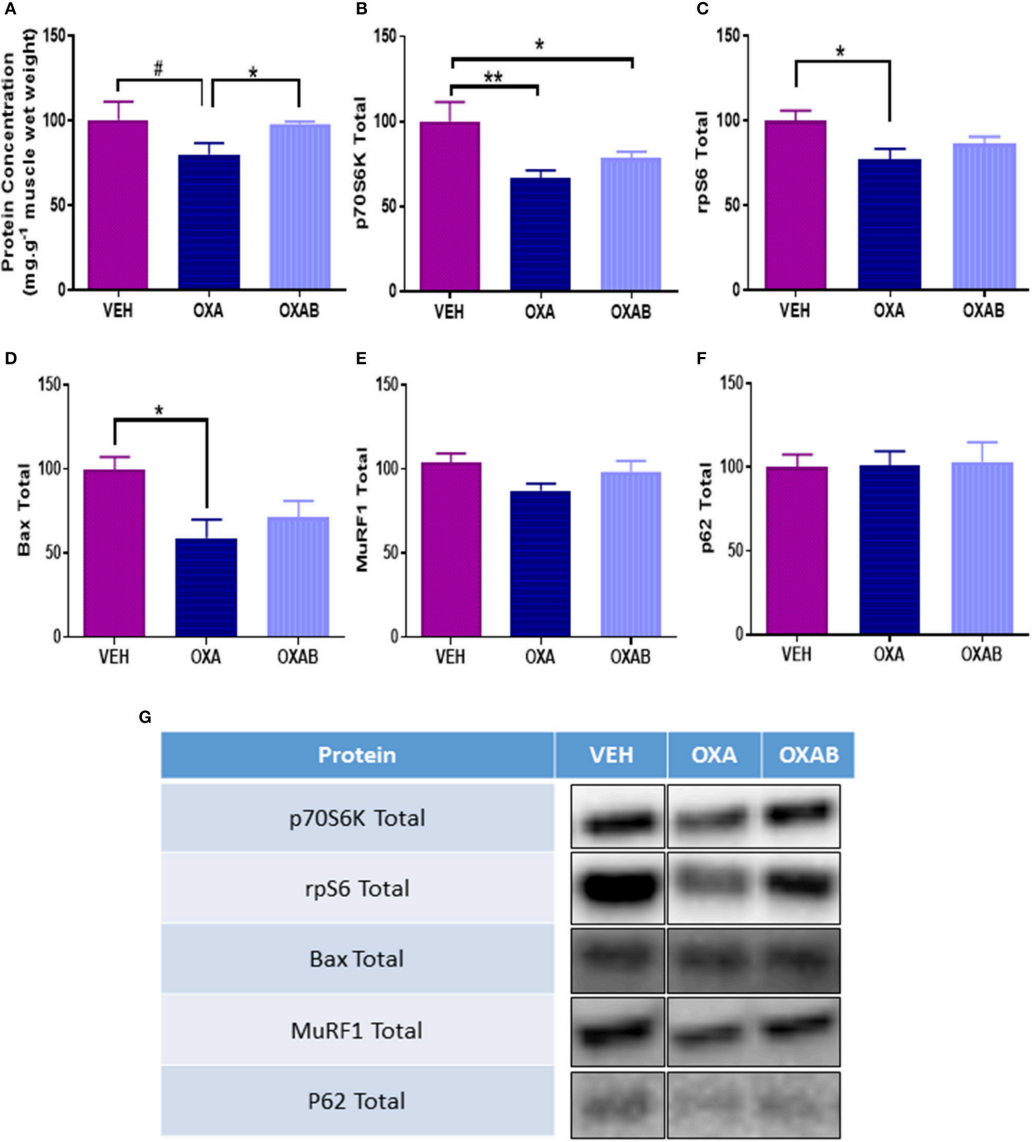
**OXA treatment reduces protein synthesis and concentration within TA muscle. (A)** OXA treatment showed a trend to reduce total protein concentration, and reduced **(B)** total p70S6K and **(C)** total rpS6 when compared to VEH suggesting a suppression of protein synthesis. **(D)** Apoptosis initiation marker total Bax was also supressed in TA muscle. Contrastingly no change in **(E)** total ubiquitin-proteasome marker MuRF1 or **(F)** autophagy marker p62 expression was noted. **(G)** Western blot representative images are digitally cut together to remove blots of other chemotherapies not presented in this manuscript, no other alteration was performed. Significance: ^*^p < 0.05, ^**^p < 0.005 Trend: ^#^*p* = 0.081. *n* = 6–8.

To elucidate whether the reduction in lean mass observed with OXA treatment corresponded with the activation of skeletal muscle atrophy signaling pathways, a variety of atrophy-related molecular signaling proteins were quantified via western blot. Of note, OXA treatment induced a 28% reduction of total p70S6K expression compared to VEH (*p* < 0.005, Figure 9B) indicating a reduced potential for protein synthesis. This was corroborated downstream by a drop in ribosomal protein S6 expression (rpS6, downstream transcription protein regulator, *p* < 0.05, Figure 9C) and a trend for a reduction in protein concentration within the TA (*p* = 0.081, Figure 9A). Of note, OXA treatment reduced BAX expression (apoptosis initiating protein, *p* < 0.05, Figure 9E) compared to VEH suggesting a reduced propensity for apoptosis induction. BGP-15 treatment protected against protein concentration reduction, however, had no observable effect on markers of protein synthesis.

## Discussion

In this study we demonstrate that OXA chemotherapy: (1) significantly reduces body weight which is underpinned by a decline in lean tissue and liver mass; (2) does not impact voluntary exercise capacity, O_2_ consumption or energy expenditure in mice; (3) shifts fiber size distribution to favor smaller fibers in the TA whilst increasing collagen, neutral lipid, and Ca^2+^ accumulation; (4) reduces FDB fiber diameter; (5) penetrates the mitochondria and increases mitochondrial population and superoxide (${\text{O}}_{2}^{-}$) production in FDB fibers and SDH content/capacity in TA; and (6) reduces molecular markers of protein synthesis pathways with the tendency to reduce intramuscular protein content. Importantly, our data highlights a novel application for the pharmacological cytoprotectant, BGP-15, which when administered with OXA (OXAB) protected against the observed reductions in lean tissue mass and FDB fiber diameter and TA protein content, mitochondrial ${\text{O}}_{2}^{-}$ production and histopathological features of TA sections. BGP-15 adjunct therapy also increased mitochondrial pool viability and reduced the energy expenditure of mice during inactivity.

Chemotherapeutic drugs are well described as toxic antineoplastic agents capable of causing significant side effects in healthy tissues (Davies and Doroshow, 1986; Sternberg et al., 2001; Lu, 2005; Argilés et al., 2015). In skeletal muscle, this correlates with sustained dysfunction resulting in fatigue and wasting (Mantovani et al., 2003; Gilliam and St Clair, 2011; Gilliam et al., 2012; Gouspillou et al., 2015), with wasting being traditionally attributed to an imbalance between protein degradation and synthesis (Sandri, 2008). Since a decline in muscle mass is negatively associated with patient survivability, quality of life and chemotherapeutic treatment options (Talvensaari et al., 1996; Oeffinger et al., 2006; van Brussel et al., 2006; Ness et al., 2007, 2012; Scheede-Bergdahl and Jagoe, 2013), methods to protect the skeletal muscle from dysfunction and loss is of paramount importance. Here we have investigated the effect of 2 weeks of OXA treatment in 6 week old Balb/c mice and observed, as expected, a marked reduction in body weight from D8 onwards (when compared to VEH, Figure 1A). Once a cumulative dose of 9 mg/kg of OXA was reached, mice ceased to accumulate body mass which was shown to be independent of caloric intake (Figure 1B), hydration index (Figure 2C) and energy expenditure (Figure 3B). Further analysis of body composition elucidated that OXA treatment depressed total lean tissue mass with a tendency for fat mass to follow the same declination when indexed against absolute body weight (Figures 2A–C). It is interesting to speculate that the loss of lean mass (and tendency for the loss of fat mass) might be strongly correlated with OXA-induced enteric neuropathy and gastrointestinal dysfunction which has been reported previously (Stojanovska et al., 2015; McQuade et al., 2016a) and which likely limits the capacity for nutrient absorption in the small intestine. Skeletal muscle mass is strongly associated with the nutritional status of the organism (Jeejeebhoy et al., 1990; Mithal et al., 2013; Moon, 2014), whereby in times of nutrient deprivation (i.e., starvation), skeletal muscle protein synthesis pathways are inhibited and degradation pathways are activated to liberate nutritionals stores (particularly glucose and amino acids) for key physiological functions (Thissen et al., 1994; Levine and Kroemer, 2008). Our protein analyses, although a small snapshot of a complex and dynamic system, suggests that OXA treatment reduces p70s6K (Figure 9B) and rpS6 expression and has the tendency to reduce intramuscular protein content, possibly via inhibition of the master hypertrophy regulator, mTOR, which is notably inhibited by nutrient deprivation in skeletal muscle (Mammucari et al., 2008; Pasiakos et al., 2010; Zoncu et al., 2011; Laplante and Sabatini, 2012). mTOR inhibition is also typically accompanied by the induction of skeletal muscle degradative pathways (Mammucari et al., 2008; Pasiakos et al., 2010). While we show no effect of OXA on MURF1 (Figure 9F) or the autophagy regulator p62 (Figure 9G), there are several other pathways implicated in nutrient-deprivation related skeletal muscle wasting that we have not investigated (such as TRAF6 Fan and Cook, 2004; Kumar et al., 2012; Paul et al., 2012) and which may be causing the loss of lean mass observed in our study.

Promisingly, adjunct BGP-15 therapy protected mice against OXA-induced changes in lean tissue mass. We have shown that BGP-15 can protect myenteric neurons against OXA-induced insult to ameliorate gastrointestinal dysfunction (McQuade et al., 2016b)—thus it is likely that BGP-15 promotes nutrient absorption and the overall nutritional status, thus protecting the muscle mass. Interestingly, we observed a marked reduction in liver weight as a result of OXA treatment which is consistent with glycogen depletion, however this effect was exacerbated by BGP-15 treatment. The effect of BGP-15 on liver mass is curious and could be a direct effect of drug metabolism that has not been reported previously. BGP-15 adjunct therapy was also observed to increase heart size to body weight ratio (Figure 2D). While this could be indicative of cardiac hypertrophy, previous studies have shown BGP-15 to be cardio-protective against heart failure and inflammation (Szabados et al., 2000; Sarszegi et al., 2012; Sapra et al., 2014). Indeed, our exercise and VO_2_ data indicate no performance deficit following OXAB treatment, thus the increased heart weight to body weight ratio observed appears not to be detrimental to function.

Exercise therapy is an emerging treatment against chemotherapy- and cachexia-driven skeletal muscle wasting (Al-Majid and McCarthy, 2001; Smuder et al., 2011; Jarvela et al., 2012; Kavazis et al., 2014; Bredahl et al., 2016). While exercise before, during and after anti-cancer therapy is a field of research that has gained traction in the recent years, there is currently no established protocol that is prescribed in the clinic to prevent and/or treat skeletal muscle atrophy during cancer treatment (André et al., 2004; Alcindor and Beauger, 2011). For these reasons and in light of our observed reduction in lean mass, investigations into exercise tolerability and capacity were performed. Surprisingly, OXA treatment did not hinder voluntary exercise participation in mice (Figures 3D–F), nor did chemotherapy alter average exercise intensity (data not shown), duration or distance (Figures 3D–F). Interestingly though, behavioral analysis of the mice in relation to time budgeting of activities of daily living revealed that OXA-treated mice engaged in fewer long lounge periods (indicative of rest and sleep) during the 24 h analysis period, which is contradictive to the fatigue reported by chemotherapy-treated patients (Love et al., 1989; Greene et al., 1993). Given we concomitantly observed a strong trend (*p* = 0.057) for OXA-treated mice to interact with the food hopper, our data suggest that these mice might be waking more frequently to eat which is consistent with our nutrient deprivation hypothesis. As with our body composition data, BGP-15 protected against this OXA-induced behavioral change.

It is reasonable to assume that exercise could be more arduous for OXA treated mice due to the decline in lean tissue mass, however, gas exchange analysis showed comparable RQ and O_2_ consumption between VEH and OXA groups at all levels of exercise intensity, and at rest. BGP-15 adjunct therapy, however, reduced basal energy expenditure at rest (Figure 3B) and increased mouse exercise performance (reduced time spent on wheel compared to other groups Figures 3D,F with same distance outcome Figure 3E) suggesting that BGP-15 has the capacity to improve exercise efficiency, possibly due to improved mitochondrial coupling (Conley et al., 2013). Indeed, enhanced mitochondrial efficiency is a key adaptation during nutrient deprivation to increase energy extraction from macronutrients in the form of ATP as opposed to heat (Weyer et al., 2000). While we did not measure skeletal muscle uncoupling protein expression or inducible uncoupling in our study (a noteworthy limitation), our data suggests that BGP-15 might protect the skeletal muscle from chemotherapy/nutrient-deprivation-induced wasting by enhancing mitochondrial efficiency (Figure 8C).

In addition to mass, muscle quality is a major contributor to the overall functional and reparative capacity of the muscle. To this effect, a reduction in quality (i.e., increased intra- and inter-muscle fat) irrespective of absolute mass would greatly impede a patients' ability to perform activities of daily living, ultimately reducing independence and quality of life. Histological analysis of TA muscles following OXA treatment highlighted features consistent with myopathy including a significant accumulation of both Ca^2+^ and neutral lipids within the TA architecture (Figures 5A,C). Furthermore, markedly higher levels of collagen deposition were identified in OXA-treated TA muscle. These findings, taken together with the fact that hind limb muscle weights were comparable to VEH following OXA treatment, suggest that gross measurement of muscle weight was not sensitive enough to detect changes in muscle mass, whereby increased collagen and fat would contribute to muscle weight. At the cross sectional level, OXA treatment shifted the fiber size distribution to the left, in particular, reducing the number of fibers within the 1,200–2,999 μm^2^ fiber cross sectional area range, whilst increasing the number of smaller fibers within the 300–1,199 μm^2^ bin ranges (Figure 4B). While the mechanism behind this fiber size distribution shift remains unclear, the significant increase in fibers above 4,900 μm^2^ in OXA treated mice (21x greater compared to VEH) suggests that OXA treatment induced pathological changes. Pseudohypertrophic fibers are a pronounced feature of myopathy, in which healthy fibers hypertrophy to compensate for the loss of strength induced by muscle atrophy, wasting and/or replacement with non-functional tissue (such as fat and connective tissue; Briguet et al., 2004; Timpani et al., 2016). Importantly, BGP-15 adjunct therapy ameliorated all of these OXA-induced pathologies by protecting against Ca^2+^, lipid and collagen accumulation and restoring a normal fiber distribution. These findings, albeit novel in the chemotherapy/skeletal muscle arena, align with the findings of a recent study by Salah et al. (2016) who demonstrated that BGP-15 could protect against fibrotic tissue and collagen accumulation within mechanically-ventilated diaphragm muscle. They suggested that BGP-15's efficacy was mediated via membrane lipid therapy mechanisms (Salah et al., 2016), an effect that has also been established in other myopathies (Gehrig et al., 2012). Furthermore, BGP-15 has recently been shown to improve certain myopathological aspects of DMD such as collagen deposition (Kennedy et al., 2016), which is consistent with our data.

Chemotherapy-induced oxidative stress has previously been linked, albeit almost exclusively following anthracycline treatment (Gilliam et al., 2012; Gouspillou et al., 2015), to mitochondrial-mediated cell damage pathways within skeletal muscle (Davies and Doroshow, 1986; Doroshow and Davies, 1986; Gilliam et al., 2012; Min et al., 2015). Since OXA, via the Pt component of the molecule, adducts nDNA resulting in DNA damage, impeded transcription/translation and cell death (Raymond et al., 1998; André et al., 2004; Gourdier et al., 2004; Alcindor and Beauger, 2011), we have previously hypothesized that OXA could induce the same damage to mtDNA (Sorensen et al., 2016). To substantiate this, atomic absorption spectrophotometry was utilized to detect Pt in the subcellular mitochondrial fraction. Pertinently, we are the first to show that OXA does in fact penetrate both the skeletal muscle and the mitochondria, suggesting that OXA could also be inducing Pt adducts into mtDNA (Figure 6B). The net effect of OXA treatment at the mitochondrial level was increased succinate dehydrogenase (SDH) content within the muscle, indicative of a greater mitochondrial oxidative capacity. However, there was a trend for OXA (*p* = 0.07) to reduce mitochondrial viability relative to VEH (Figure 8C), highlighting that the innate response to oxidative stress and/or mitochondrial toxicity is to increase the total pool to preserve energy production capacity (Hsin-Chen et al., 2000) as indicated by more intense SDH staining (Figure 6C) and mitochondrial density (Figure 8C). Consistent with this finding, a marked increase in mtROS production was detected in FDB fibers following OXA treatment (Figure 8B) giving further support to our theory that mitochondrial biogenesis could be increased in order to accommodate oxidative stress and a dysfunctional mitochondrial population (Hsin-Chen et al., 2000; Kujoth et al., 2005; Stowe and Camara, 2009; Sorensen et al., 2016). Remarkably, BGP-15 completely protected against the OXA-induced increase in mtROS production and reduction in FDB fiber diameter and intramuscular protein content, however, this effect was independent from any modulation of protein synthesis, atrophy, autophagy or apoptosis markers measured in our study (Figure 9). Moreover, BGP-15 treatment was shown to improve mitochondrial viability by 60% from OXA- and 40% from VEH-treated groups (Figure 8C), which we originally hypothesized would be due to molecular inhibition of PARP and the restoration of mitochondrial substrates (NADH) to the electron transport chain (NADH is a known substrate of PARP) (Zhou et al., 2006). Unexpectedly though, we were unable to detect any changes in PARP1, PARP2, or total PARylation with OXA or OXAB treatment (Figure 7A–C). This suggests in the first instance that OXA treatment at the dosage administered (18 mg/kg cumulative dose) does not stimulate PARP activity in skeletal muscle; and secondly that BGP-15 improved mitochondrial viability and normalized mtROS production via an alternative mechanism. Since mtROS are key mediators of muscle maintenance pathways (Powers et al., 2005, 2011, 2012; Min et al., 2011; Smuder et al., 2011), BGP-15's capacity to reduce mtROS could be key to its therapeutic benefit. By limiting oxidative stress, BGP-15 likely affords protection to the mitochondria from protein oxidation and dysfunction, subsequently resulting in the observed increase in both mitochondrial density and viability. While not a definitive mechanism, we speculate that BGP-15 might achieve this by enhancing mitochondrial coupling efficiency, which would explain the lower resting energy expenditure observed in mice and potentially reduce electron leak (and therefore ROS production) concomitantly with proton leak. Certainly, our data highlights the therapeutic potential of BGP-15 to protect against the debilitating side-effects of OXA chemotherapy, at the whole body, organ and mitochondrial levels.

## Limitations

It should be noted, that the present study is limited in its resemblance to the clinical setting in which only patients afflicted with cancer are administered chemotherapy treatment. As such, it does not take into consideration the complex interactions between tumor-mediated cachexia and chemotherapy-driven muscle wasting. Furthermore, the complexity of the pathways underlying cachexia- and chemotherapy-driven wasting have not been completely illuminated herein, with only a selection of highly investigated protein markers of molecular atrophy signaling being presented. At the organ level reductions in the liver and heart mass were observed following OXA treatment and were exacerbated with OXAB treatment. Since blood samples were not taken, we were unable to investigate markers of liver damage (such as aspartate transaminase, alanine transaminase and lactate dehydrogenase levels) which may have shed light on the underlying mechanisms behind the observed decrease in liver sizes with OXA and combination OXAB therapy. Although investigations of lower limb muscle weights showed no significant changes, this finding is limited in its interpretation as the remaining upper limb and core muscles as well as organs, such as the gastrointestinal tract and brain, were not collected. Furthermore, fiber typing of the skeletal muscle was not performed and this would be useful to determine whether the OXA-induced increases in SDH and neutral lipid density within TA sections were due to oxidative fiber type transitions. While we are currently undertaking these investigations, previous studies have shown that muscle mass loss induced by other pro-oxidant chemotherapy drugs (specifically doxorubicin) is independent of fiber type (Gilliam et al., 2009).

## Conclusion

Chemotherapy treatment remains the foremost defense against advanced neoplastic growth, however treatment often creates and exacerbates considerable dysfunctions within the skeletal muscle system, which endure well after chemotherapy treatment ceases. This study has demonstrated that OXA-treatment reduces lean mass, with the suppression of protein synthesis pathways a likely contributing mechanism. Moreover, OXA treatment induces various myopathic features including the accumulation of Ca^2+^, neutral lipid, and collagen tissue within the muscle architecture, a reduction in intramuscular protein content, a leftward shift in the fiber size distribution and exacerbated mtROS production. Importantly, we have highlighted the efficacy of BGP-15 therapy to combat OXA-driven lean mass loss. For the first time we have demonstrated that OXA can independently penetrate the mitochondria, and is thus a likely contributor to these myopathic features. We importantly show that BGP-15 adjunct therapy either completely or partially protects the skeletal muscle against these deleterious side effects. BGP-15 appears to modulate the cytoprotective response to protect the mitochondria from damage and enhance both pool density and viability, albeit not via PARP inhibition. Although the precise mechanism of action requires further elucidation, here we show a novel application for the small molecule, BGP-15, in the protection of skeletal muscle against platinum-based chemotherapy-induced myopathy and wasting.

## Author contributions

ER, AP, and AH conceived the study and obtained funding. ER, AH, JS, and CT designed the experiments. JS, CT, DC, JC, AT, VS, and MS contributed to the experimental acquisition of the data. ER, JS, AP, CT, DC, JC, VS, and MS contributed to the analysis of the data. All authors contributed to the interpretation of the data and the writing and editing of the manuscript. All authors approve of the final manuscript submission and agree to be accountable for all aspects of the work. The authors all contributed to the writing of this manuscript.

## Funding

This work was supported by the Centre for Chronic Disease and the Institute of Sport, Exercise and Active Living (ISEAL) Clinical Exercise Program funding schemes (both Victoria University).

### Conflict of interest statement

The authors declare that the research was conducted in the absence of any commercial or financial relationships that could be construed as a potential conflict of interest.

## Supplementary material

Supplementary Figure 1 **Stain free images of western blot analysis**. Representative images for PARylation and PARP1 and PARP2 total used for signal protein analysis in the range of ~250–25 kDa.

Supplementary Figure 2 **Stain free images of western blot analysis**. Representative images used for signal protein analysis in the range of ~250–25 kDa.
