# Supplementary figures and images for: BGP-15 Protects against Oxaliplatin-Induced Skeletal Myopathy and Mitochondrial Reactive Oxygen Species Production in Mice
Source: Front Pharmacol. 2017 Apr 10;8:137. doi: 10.3389/fphar.2017.00137 (PMC5385327; doi:10.3389/fphar.2017.00137)

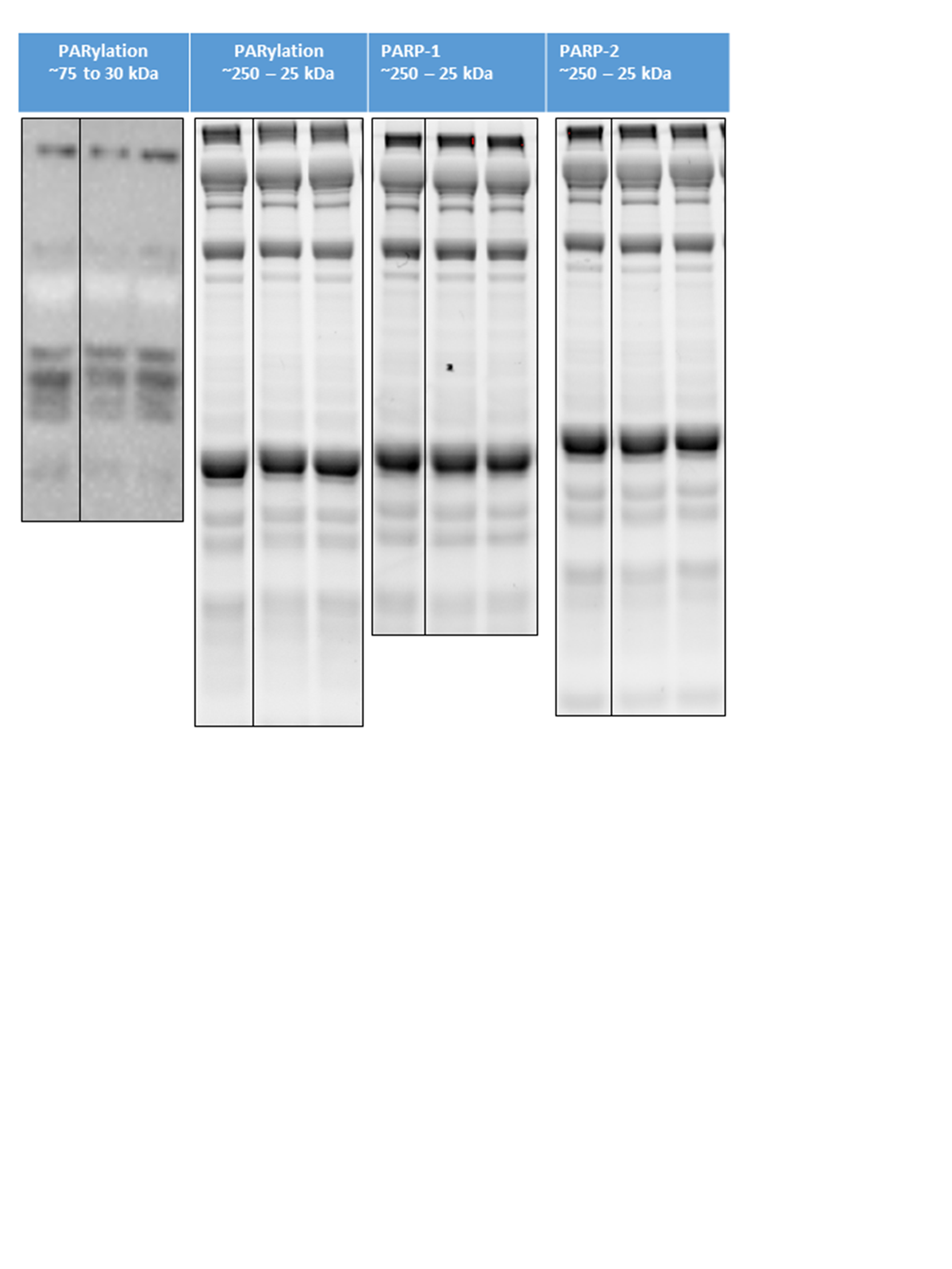

Supplement: Supplementary Figure 1 — Stain free images of western blot analysis. Representative images for PARylation and PARP1 and PARP2 total used for signal protein analysis in the range of ~250–25 kDa. [file Image1.tif]

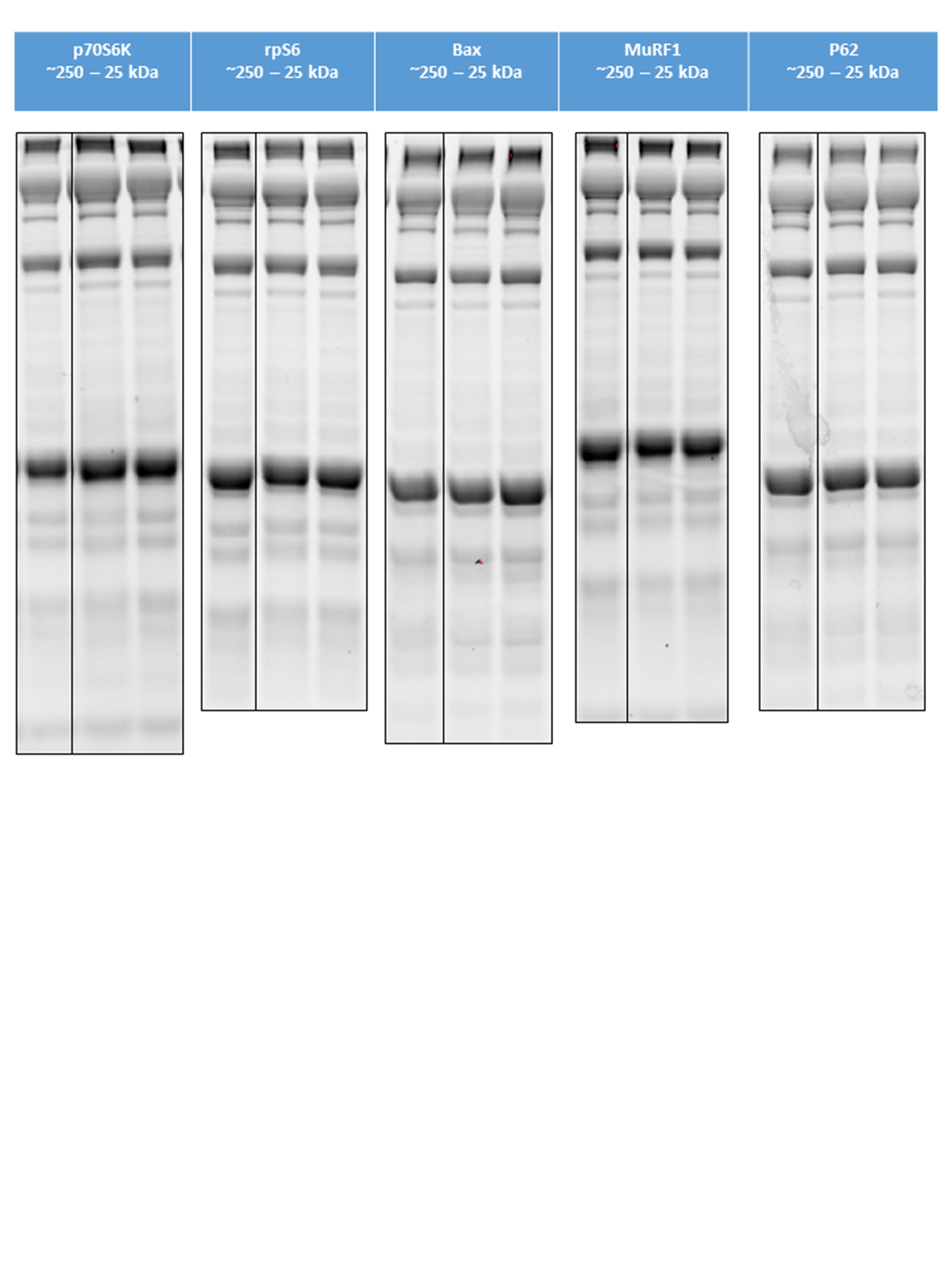

Supplement: Supplementary Figure 2 — Stain free images of western blot analysis. Representative images used for signal protein analysis in the range of ~250–25 kDa. [file Image2.tif]
